# The American and Colonial Journals: Pathology, Practical Medicine, and Therapeutics

**Published:** 1840-01

**Authors:** 


### II. THE AMERICAN AND COLONIAL JOURNALS.

### PATHOLOGY, PRACTICAL MEDICINE, AND THERAPEUTICS.

*On the use of Arsenic in Chorea.* By D. M. Reese, M D., Professor of Medicine, Albany College, New York.

These results of experience have led me successively to test the comparative merits of the various other remedies which have been reported as successful, as the rubigo ferri, cuprum ammoniacum, sulphate and flowers of zinc, oxide of bismuth, camphor, electricity, nitrate of silver, &c., and after very considerable opportunities, I have learned to rely upon the tonic powers of *arsenic* in preference to any and all other medicines of this class; and having never known it to fail in effecting a radical and permanent cure, I feel great confidence in recommending it to the profession in all those cases in which the tonic plan is decided upon, either with or without preliminary medication. The arseniate of potash, as existing in the formula of Fowler's solution, is the preferable mode of exhibition, and its dose should be graduated according to the ability of the stomach to receive it without nausea. In the most numerous subjects, varying from seven to sixteen years of age, six or eight drops should be given night and morning, gradually increasing its dose and frequency. Adults may take ten drops increasing to fifteen, and even twenty, three times a day, and in all cases it should be persevered in for a week or more after all the spasmodic symptoms have disappeared. The only unpleasant effects are nausea or vomiting if the dose be too large, and occasionally a tumefaction of the head and face if too long persisted in. On either of these effects being produced the medicine should be discontinued for a few days, and then resumed in a diminished dose. With these precautions, I have, for a number of years, employed this remedy in over two hundred cases of chorea, without ever having witnessed any of the untoward results upon the constitution, said to follow the exhibition of arsenical preparations. So far from any of the sequelæ, which are apprehended by the alarmists, my patients have, under its use, not only recovered from chorea, but many of them seem to have since acquired vigorous constitutions and improved health. I have used it in infancy, in delicate females during pregnancy, and under circumstances supposed to forbid its employment by many, and I have never once seen cause to regret its exhibition.

But while I thus dwell upon the value of arsenic as a remedial agent in chorea, I have not failed to superadd auxiliary treatment, and hence nutritious diet, active exercise, cordial drinks, and the cold bath, both plunging and showering, have all been usefully employed. And, in the examples, comparatively few, in which counter-irritation has been indicated, I have obtained most favorable results by the aid of rubefacients, blisters, setons, issues, and the eruption of the tartrite of antimony, applied to the spine. But I have never had occasion for active depletion even by purgatives, although when constipation exists in connexion with chorea, or where there has been present the suspicion of worms, I have interposed a cathartic or anthelmintic, while, at the same time, persevering in the tonic treatment. *New York Journal of Med. and Sur. Oct.* 1839.

